# Relationship between *Helicobacter pylori* infection and obesity in Chinese adults: A systematic review with meta-analysis

**DOI:** 10.1371/journal.pone.0221076

**Published:** 2019-09-11

**Authors:** Xinlan Xu, Weide Li, Lan Qin, Wenjiao Yang, Guowei Yu, Qishan Wei

**Affiliations:** 1 School of Mathematics and Statistics, Lanzhou University, Lanzhou, Gansu, China; 2 School of Public Health, Lanzhou University, Lanzhou, Gansu, China; 3 Medical College of Northwest University for Nationalities, Lanzhou, Gansu, China; 4 Maternal and Child Health Hospital, Lanzhou, Gansu, China; Faculty of Pharmacy, Ain Shams University, EGYPT

## Abstract

**Background:**

Obesity is highly prevalent worldwide. More and more studies have been conducted on the relationship between *H*. *pylori* infection and obesity or overweight. But the relationship between them is controversial in the literatures and there is no comprehensive evidence for the correlation.

**Aim:**

To evaluate the prevalence of *H*. *pylori* infection in Chinese adult subjects who received routine physical examinations and the relationship between *H*. *pylori* and obesity.

**Methods:**

Literatures on *H*. *pylori* infection and obesity in Chinese population were searched in online databases. Relevant data were extracted independently by two researchers and meta-analysis was performed by using Review manager 5.3 software.

**Results:**

22 articles were selected with a total sample size of 178033. The pooled prevalence of *H*. *pylori* was 42% (95%CI: 37% to 47%) and mean difference of BMI between subjects with and without *H*. *pylori* infection was 0.94 (95%CI: -0.04 to 1.91). 9 eligible studies with 27111 subjects were used to calculated pooled OR value because they contained obesity groups. The OR value showed that *H*. *pylori*-positive subjects tended to be obese at a risk of 1.20 (95% CI: 1.13 to 1.28).

**Conclusion:**

In China, obesity has association with *H*. *pylori* infection. *H*. *pylori* infection may be one of the risk factors for obesity.

## Introduction

Obesity has become a health problem of global concern and its prevalence is on the rise. The World Health Organization (WHO) classifies people as overweight (25<BMI<30) and obese (BMI>30) according to their body mass index (BMI, kg/m^2^) which is calculated as one’s body weight (kg) divided by his squared body height (m^2^) [1]. Another classification for Asian populations was used [overweight: 24<BMI<28, obesity: BMI>28] [2]. A study published in the Lancet reported that the proportion of overweight or obese adults was 37% in men and 38% in women in 2013, and it had increased since 1980 [3]. Meanwhile, the prevalence of obesity in developed country is higher than that in developing country. Although obese people have longer life expectancy than before due to better health care and risk factor management received by them, obesity complications bring them more burden, such as type 2 diabetes, hypertension, chronic kidney disease, fatty liver disease and so on [4–6].

Recently, many studies indicated that *Helicobacter pylori* (*H*. *pylori*, Hp) had relationship with obesity [7, 8]. *H*. *pylori* is a gram-negative, bacillus bacterium which colonizes the human stomach and was began to be known to the world since 1984 [9]. *H*. *pylori* infected almost half of people worldwide and the number of infected people was 4.4 billion in 2015 [10]. A recent meta-analysis showed that the global prevalence of *H*. *pylori* was near 44.3%, and was 42.7% in females while 46.3% in males [10]. The *H*. *pylori* infection rate varies in different region which is 50.8% in developing countries while 34.7% in developed country [11]. About one-third of adults are still infected in north European and North American populations, whereas in south and east Europe, South America and Asia, the prevalence of *H*. *pylori* is often higher than 50% [12].

This kind of bacterial pathogen is well recognized as one of the main cause of peptic ulcer disease. The organism has also been thought to be a major risk factor for gastric cancer, colon cancer and mucosa-associated lymphoid tissue lymphoma. [13–15] *Helicobacter pylori* infection usually lasts for life after its first establishing.

Several epidemiological studies have focused on the correlation between *H*. *pylori* colonization and BMI and obesity. The results of these studies got contrasting results. Some studies showed that BMI of patients with *H*. *pylori* infection was higher than that without [8]. A cohort study in Israel reported *H*. *pylori* infection rate was higher in obese subjects than that in normal weight ones [16]. A review showed negative correlation existing between the prevalence of *H*. *pylori* and obesity in developed countries [17]. For all we know, the reviews of relationship between *H*. *pylori* infection and overweight or obesity among Chinese have not been reported previously. Thus, in the present review, we choose studies reporting Chinese subjects and we aimed: i) to assess the prevalence of *H*. *pylori* in China; ii) to examine mean differences in body mass index (BMI) and other factors across groups with or without *H*. *pylori* infection and evaluate the risk of *H*. *pylori* infection to obesity.

## Materials and methods

### Search strategy

Six electronic databases including PubMed, EMBASE, Cochrane Library, Web of Science and two Chinese databases, CNKI (China National Knowledge Infrastructure) and WanFang, were searched from their establishment to December 2018 using the following search strategies (Table 1) which included keywords related to *H*. *pylori*, obesity and country. The same kind of terms were connected by the Boolean operator “OR” and different kinds of terms were connected by “AND”.

**Table 1:** Study keywords in search strategy.

#1 *Helicobacter pylori*[MeSH Terms]) OR ("*Helicobacter pylori*"[Title/Abstract] OR "*H*. *pylori*"[Title/Abstract] OR "*Campylobacter pylori*"[Title/Abstract] OR Hp[Title/Abstract]
#2 (Obesity[MeSH Terms]) OR (obesity[Title/Abstract] OR obese[Title/Abstract] OR adiposity[Title/Abstract] OR adipose[Title/Abstract] OR overweight[Title/Abstract] OR fatness[Title/Abstract] OR BMI[Title/Abstract] OR "body mass index"[Title/Abstract] OR "weight gain"[Title/Abstract] OR "weight loss"[Title/Abstract] OR "Body weight"[Title/Abstract] OR "body weight changes"[Title/Abstract] OR "over weight"[Title/Abstract])
#3 ((China[MeSH Terms]) OR China[Title/Abstract]) OR Chinese[Title/Abstract]
#1 AND #2 AND #3

The inclusion criteria were: (1) the subjects of the study were people who underwent physical examinations; (2) the demographic data of the subjects in the literature were complete, and the sample sizes of both the *H*. *pylori*-positive group and the *H*. *pylori*-negative group, as well as overweight or obesity in each group, or the mean and standard deviation of BMI in each group can be obtained. Studies were excluded if: (1) they were articles such as conferences and reviews; (2) they were about pathological analysis or animal experiments; (3) the samples or contents of them were repeated or very similar to others.

### Data abstraction

Two investigators reviewed all literature independently and retrieved studies according to inclusion or exclusion criteria. Any disagreement will be determined by discussion participated by a third investigator. Data extraction was carried out from literatures meeting inclusion criteria, mainly including the following contents: authors, publish year, survey year, survey region and methods to diagnose *H*. *pylori*, demographic of subjects, prevalence of *H*. *pylori*, number of cases with overweight or obesity or the mean and standard deviation of BMI in population with and without *H*. *pylori* infection. The eligibility of relevant studies was evaluated using the cross-sectional study quality evaluation criteria recommended by the Agency for Healthcare Research and Quality (AHRQ) [18]. There are 11 items in the evaluation criteria, including the selection of subjects, quality control and data processing, and the answers are "yes", "no" and "unclear". An item would be scored “0” if it was answered “no” or “unclear”; if it was answered “yes”, then the item scored “1”. Article quality was assessed as follows: low quality = 0–3; moderate quality = 4–7; high quality = 8-11. The process was independently conducted by two researchers at the same time. In case of disagreement, the dispute shall be discussed and decided by a third investigator.

### Statistical analysis

The Review Manager 5.3 software was adopted for meta-analysis of prevalence, mean difference and the odds ratio. The results of continuous data were represented by weighted mean difference (WMD) and 95%CI, and the results of classified data were represented by OR and 95%CI. The heterogeneity between studies was determined by I^2^ test. If I^2^ > 50%, the random effect model was adopted; if I^2^ < 50%, the fixed effect model was adopted. To ensure the stability of meta-analysis results, sensitivity analysis was performed, removing one at a time to compare whether there were significant differences in effect values before and after removal. And the publication bias assessment was conducted on the included literature. If the plot was symmetric, it was considered that there was no publication bias.

## Results

**Fig 1:**
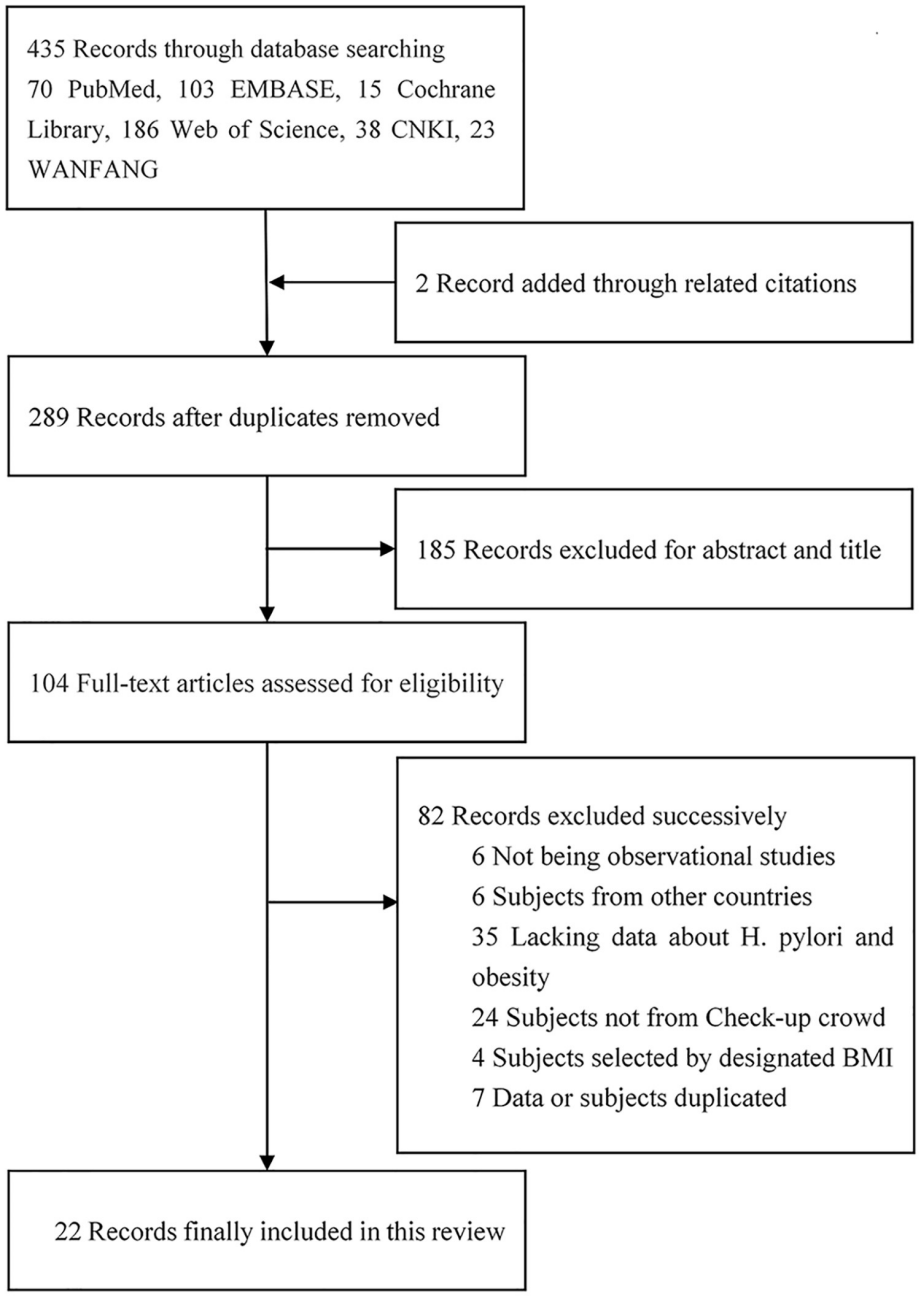
Literature retrieval flow chart.

The literature selection process is shown in Fig 1. Of 435 potentially relevant articles (2 articles were added through related citations), 289 relevant articles left after removing duplicates, 185 articles were then excluded due to their titles and abstracts not meeting inclusion criteria. Subsequently, 43 articles were excluded due to the following reasons: not observational studies (n = 6), subjects included did not came from China (n = 6), lacking data about *H*. *pylori* and BMI (n = 35), subjects not from Check-up crowd (n = 24), subjects selected by designated BMI (n = 4), data or subjects duplicated (n = 7). Finally, twenty-two observational studies (all cross-sectional studies) met all inclusion criteria. The characteristics and quality assessment of the included cross-sectional studies are presented in Table 2. Totally 178033 subjects involved in this review and 70761 of them infected with *H*. *pylori*. Age of studied subjects ranged from 17 years to 91 years or elder age. Test methods of *H*. *pylori* infection used in these studies were urea breath test (n = 15), serum diagnosis method through *H*. *pylori*-specific IgG antibody (n = 5), rapid urease test (n = 5), biopsy or histology method (n = 1). Stool antigen test was not used in any study.

**Table 2:** Characteristics of the different cross-sectional studies.

Study ID	Survey Year	Method	City	Age	*H*. *pylori* prevalence (%)	Related target	Score
01 Wu FJ 2018 [19]	2016–2017	UBT	Zhengzhou	48	200/600(0.33)	MS	7
02 Wan ZC 2018[20]	2016	UBT	Wuhan	43	1687/5168(0.33)	HTN	9
03 Wei WZ 2018[21]	2016	UBT	Shijiazhuang	20–91	861/1613(0.53)	BL	8
04 Wu YH 2017[22]	2016	UBT	Qianjiang	≥18	353/823(0.43)	BMI	8
05 Gao Y 2018[23]	2015–2016	UBT	Wuhan	≥20	2304/6869(0.35)	BL	8
06 Chen LW 2018[24]	2014–2016	UBT	Taiwan	>30	1358/2604(0.52)	obesity	10
07 Yang W 2016[25]	2014	RUT	Shuozhou	73.2	80/191(0.42)	MS	9
08 Li H 2016[26]	2014	UBT	Zhejiang	47	3732/8308(0.45)	FLD	9
09 Fan N.2018[27]	2013–2014	UBT	Shanghai	48.3	17323/28171(0.61)	NAFLD	9
10 Kong XL 2017[28]	2013–2014	S	Jinan	48.6	4541/22044(0.21)	CKD	10
11 Sun Y 2016[29]	2013–2014	UBT	Shanghai	46.1	9836/22103(0.45)	BL	9
12 Xu CF 2014[30]	2013	UBT	Zhejiang	46	3859/8820(0.44)	BMI	8
13 Han X 2016[31]	2013	UBT	Shiyan	64	15295/30810(0.5)	T2MD	9
14 Ma ZH 2013[32]	2012	S	Beijing	48	1492/3085(0.48)	BL	9
15 Xu MY 2017[33]	2012–2016	S	Beijing	45	7804/17791(0.44)	anemia	9
16 Song Y 2015[34]	2011–2013	UBT	Guiyang	21–84	508/1107(0.46)	BL	8
17 Liu A 2013[35]	2011.1–12	UBT	Beijing	47	3481/11514(0.3)	HbA1c	8
18 Lei YH 2017[36]	2011	UBT	Wuhan	70.5	134/427(0.31)	MS	8
19 Zhang Y 2015[37]	2010–2012	UBT	Wuhan	52.2	839/2050(0.41)	obesity	9
20 Yang GH 2014[38]	2000–2009	B	Taiwan	≥60	182/324(0.56)	obesity	10
21 Ran L 2011[39]	2009.	S	Chongqing	21–65	651/2188(0.3)	/	8
22 Zou D 2011[40]	2007–2008	S	Shanghai	50	733/1022(0.72)	GD	10

UBT: urea breath test; RUT: rapid urease test; S: Serology; B: biopsy or histology; BMI: body mass index; FLD: fatty liver disease; NAFLD: Nonalcoholic fatty liver disease; BL: Blood lipid; HTN: hypertension MS: metabolic syndrome HbA1c: glycosylated hemoglobin levels; CKD: chronic kidney disease; GD: gastrointestinal disease.

### The prevalence of *H*. *pylori* in different subgroups

All included studies were used in the analyses to assess the prevalence of *H*. *pylori*. The prevalence of *H*. *pylori* among all subjects was 42% (95%CI: 37% to 47%), as shown in Table 3. Subgroup analysis according to test methods and regions was also performed on 22 studies that provided prevalence on adult subjects. The results showed that the pooled detection rate of urea breath test method was higher than that of other methods (serology, biopsy, rapid urease test), and the detection rate of *H*. *pylori* in first-tier cities (Beijing, Shanghai) was higher than that in other cities. Stratified analysis was also carried out for subset of those 22 studies which contained stratified information of gender, age and body type. The prevalence of *H*. *pylori* in elderly population was higher than it in the middle-aged population and was lowest in the young population. Obese population had highest *H*. *pylori* infection rate of 51% (95CI: 42% to 59%) in three body shape groups. After grouping analysis, there was still high heterogeneity, and the data were processed by random effect model. Due to the limited information provided in included literatures, more subgroup analysis were failed to be conducted.

**Table 3:** Prevalence of *H*. *pylori* infection in different subgroups.

Group		Article N	Total subjects N	Hp(+) N	Prevalence of *H*. *pylori*, 95% CI
**Total**		22	178033	70761	0.42 [0.37, 0.47]
**Test method**		22	178033	70761	0.42 [0.37, 0.47]
	UBT	15	130987	55278	0.41 [0.38, 0.45]
	Other	7	47046	15483	0.44 [0.32, 0.56]
**Region**		22	178033	70761	0.42 [0.37, 0.47]
	First-tier city	6	84087	34177	0.45 [0.39, 0.51]
	Other city	16	93946	36584	0.41 [0.34, 0.48]
**Gender**		18	167310	66612	0.42 [0.38, 0.46]
	Male	18	96571	38234	0.44 [0.38, 0.49]
	Female	18	70739	28378	0.41 [0.35, 0.46]
**Age**		5	15475	5181	0.47 [0.38, 0.56]
	≤40	3	3496	1039	0.42 [0.24, 0.60]
	40–60	3	8976	3081	0.46 [0.24, 0.67]
	≥60	5	3003	1220	0.51 [0.32, 0.70]
**Body shape**		9	42490	17354	0.47 [0.43, 0.51]
	Normal	8	21540	8489	0.43 [0.37, 0.50]
	Overweight	8	15449	6497	0.47 [0.39, 0.55]
	Obese	9	5326	2294	0.51 [0.42, 0.59]

### Estimated differences in BMI between subjects with and without *H*. *pylori* infection

As shown in Table 4, main anthropometric and biochemical characteristics per *H*. *pylori* group at baseline were provided in parts of included studies. For original data which presented as median, interquartile, sample mean and standard deviation were estimated from Luo [41], Wan [42]. Mean differences of the physiological and biochemical indexes were estimated, concluded in Table 5. The average HDL-C of the Hp-positive group was lower than that of the *H*. *pylori* negative group, but the BMI, age, SBP, DBP, TG, TC and LDL-C of the *H*. *pylori* positive group were all higher than that of the negative group. There was still great heterogeneity in the study corresponding to each index.

**Table 4:** Main anthropometric and biochemical characteristics per *H*. *pylori* group at baseline provided in part of included studies.

Study ID		Age	BMI (kg/m2)	SBP (mmHg)	DBP (mmHg)	TG (mmol/L)	TC (mmom/L)	HDL-c (mmom/L)	LDL-c (mmom/L)
01	Hp(+)	50±12.6	26.2±2.73	138±11	86±11	2.1±1.31	5.1±1.11	1.3±0.22	3.6±1.12
	Hp(-)	48±13.6	23.3±2.21	125±12	75±10	1.1±0.76	4.9±1.22	1.6±0.21	3.1±0.88
02	Hp(+)	44±11.9	24.2±3.34	124±18	76±12	1.5±1.1	4.6±0.87	1.2±0.3	2.8±0.74
	Hp(-)	42±12.1	23.6±3.38	122±16	75±11	1.5±1.17	4.6±0.87	1.2±0.3	2.7±0.75
03	Hp(+)	NA	25.3±4.3	NA	NA	1.37±1.1	4.83±1.25	1.39±0.46	2.69±1.05
	Hp(-)	NA	25.4±4.4	NA	NA	1.33±1.03	4.92±1.21	1.43±0.47	2.76±0.99
07	Hp(+)	75±10.8	24.3±2.7	132±14	76±10	1.2±0.52	4.4±0.88	NA	NA
	Hp(-)	72±11.1	23.1±2.74	133±13	74±8	1.3±0.81	4.2±1.15	NA	NA
08	Hp(+)	47±10.8	24.1±3.2	127±18	78±11	1.4±0.73	4.9±0.92	1.1±0.28	2.6±0.64
	Hp(-)	47±11.6	23.8±3.19	127±18	77±12	1.4±0.79	4.8±0.93	1.1±0.29	2.6±0.63
09	Hp(+)	48±14.9	23.9±3.3	128±20	74±12	1.5±1.2	4.8±0.9	1.4±0.4	3±0.8
	Hp(-)	48±15.1	23.5±3.2	126±19	73±11	1.4±1.2	4.8±0.9	1.5±0.4	2.9±0.8
10	Hp(+)	52±14	25.8±3.5	133±20	80±12	1.5±1.15	5±0.94	1.4±0.25	3±0.73
	Hp(-)	48±14.3	25.3±3.6	130±19	78±12	1.5±1.25	5±0.97	1.4±0.25	3±0.37
11	Hp(+)	NA	NA	NA	NA	1.70±1.43	5.08±0.975	1.31±0.32	2.99±0.80
	Hp(-)	NA	NA	NA	NA	1.61±1.32	0.06±0.98	1.35±0.38	2.96±0.79
12.	Hp(+)	46±9.6	24±3.3	126±17	78±11	1.4±0.73	4.8±0.88	1.1±0.27	2.6±0.61
	Hp(-)	46±11.1	23.7±3.18	126±18	77±12	1.3±0.78	4.8±0.88	1.1±0.28	2.5±0.59
13	Hp(+)	64±8.6	24.3±3.36	140±23	80±13	1.5±1.07	4.8±1.12	1.5±0.44	2.7±0.87
	Hp(-)	65±8.3	24.3±3.36	139±22	79±12	1.5±1.06	4.7±1.14	1.5±0.46	2.7±0.89
14	Hp(+)	50±18.9	24.5±3.27	NA	NA	5±0.92	1.6±1.26	1±0.21	2.8±0.69
	Hp(-)	47±19.2	24.3±3.35	NA	NA	4.9±0.92	1.5±1.3	1±0.22	2.7±0.73
18	Hp(+)	71±7.9	24.6±2.87	131±16	78±10	1.9±1.51	4.9±0.85	1.4±0.37	2.7±0.77
	Hp(-)	70±7.4	24.1±3.2	132±16	79±9	1.4±0.72	4.9±0.85	1.6±0.47	2.6±0.72
19	Hp(+)	52±11.3	25.3±3.36	125±13	77±10	1.5±0.91	4.8±0.88	1.2±0.3	2.9±0.83
	Hp(-)	53±11.3	25±3.04	124±13	78±11	1.5±0.84	4.7±0.91	1.2±0.29	2.8±0.76
20	Hp(+)	67±5.3	25.1±3.6	NA	NA	NA	NA	NA	NA
	Hp(-)	68±5.8	24.4±3.3	NA	NA	NA	NA	NA	NA

BMI, body mass index; SBP, systolic blood pressure; DBP, diastolic blood pressure; TG, triglycerides; TC, total cholesterol; HDL-C, high-density lipoprotein cholesterol; LDL-C, low-density lipoprotein cholesterol; Hp, *Helicobacter pylori*; N, number. Data are presented as numbers or mean ± standard deviation.

**Table 5:** Summary of mean differences on anthropometric and biochemical characteristics between Hp (+) and Hp (-) groups.

Variable	Number of study	Mean difference (95% CI), %	Q Statistics	I^2^ (%)
BMI	12	0.94 [-0.04, 1.91]**	220	95
Age	12	0.60 [0.38, 0.81]	367	97
SBP	10	2.12 [0.83, 3.41] **	225	96
DBP	10	1.42 [0.70, 2.14] **	150	94
TG	11	0.10 [0.05, 0.16] **	167	94
TC	11	0.04 [0.01, 0.07] **	33	70
HDLC	10	-0.06 [-0.09, -0.04] **	450	98
LDLC	10	0.06 [0.03, 0.09] **	75	88

** p < 0.01 between Hp(+) group and Hp(-) group

### Meta-analysis of the impact of *H*. *pylori* on obesity

**Fig 2:**
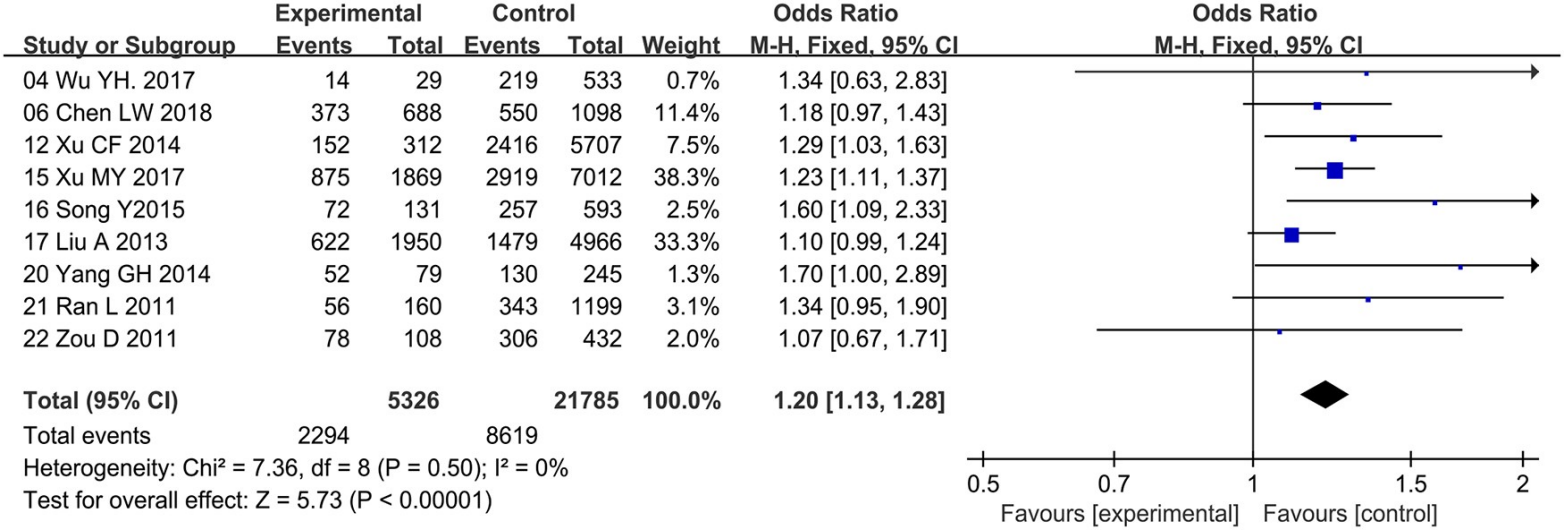
Forest plot of the odds ratio with corresponding 95% confidence intervals for *H*. *pylori* infection on obese subjects versus normal weight subjects. Size of squares reflect the statistical weight of each study. Pooled OR value is indicated by an unshaded diamond.

**Fig 3:**
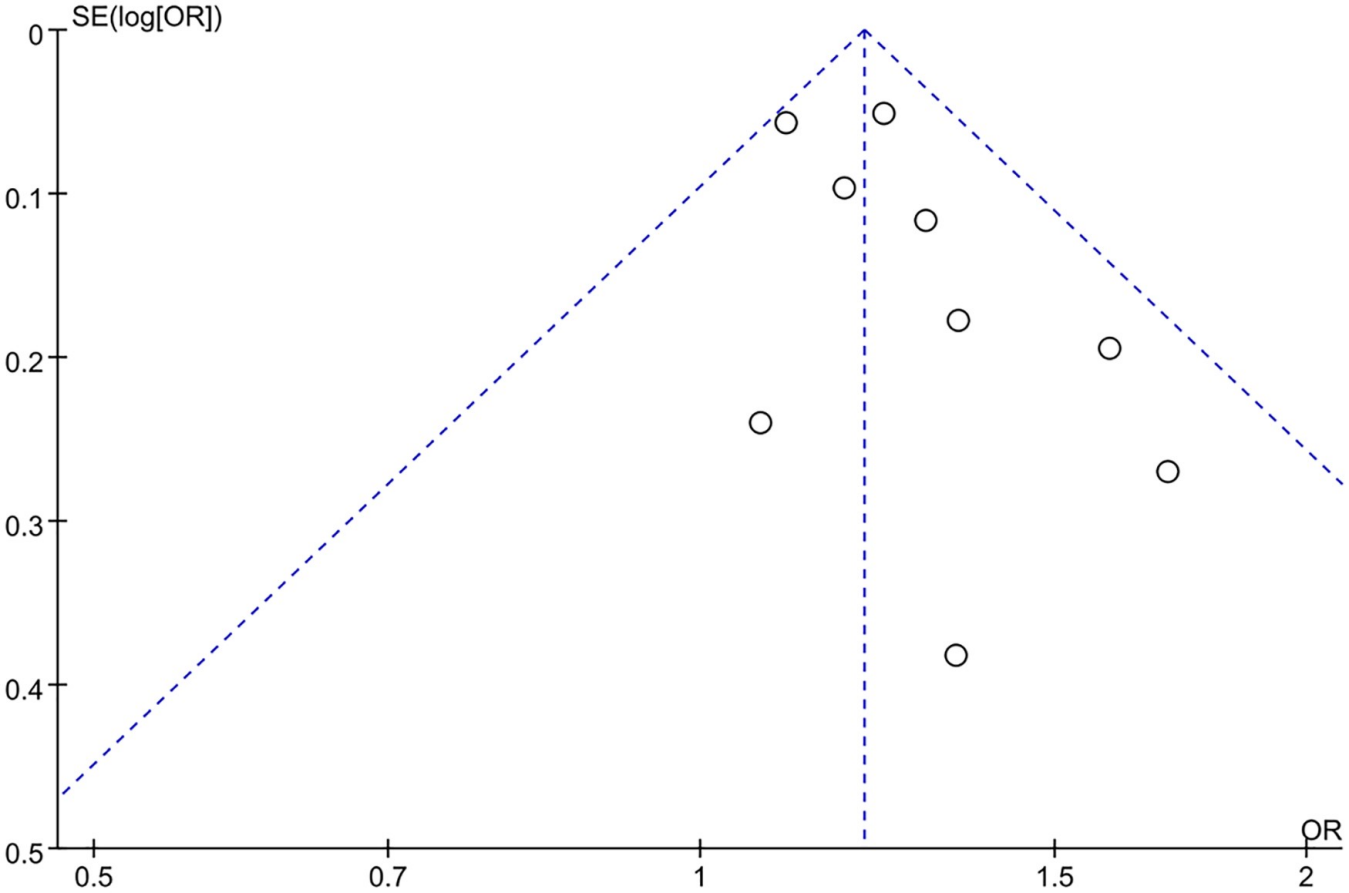
Funnel plot of the association between *H*. *pylori* infection and obesity.

Nine references were included for this meta-analysis, because only in these studies, we could get case number of patients with *H*. *pylori* in both obesity and normal weight groups. There was no statistical heterogeneity being found (I^2^ = 0.0%), so we selected a fixed-effect model for this analysis (Fig 2). The population size, demographic data, and the *H*. *pylori* infection rate for this subset can be queried by their name in Table 2. The results showed that the *H*. *pylori* infection rate in the obese patients group was lower than that in the normal-weighted control group (OR = 1.20, 95% CI: 1.13 to 1.28). It shows that there is no significant publication bias in this study (see Fig 3 for funnel plot).

## Discussion

This study is the first systematic review and meta-analysis about the relationship of *H*. *pylori* infection and obesity in China. The estimated prevalence of *H*. *pylori* in Chinese adult subjects receiving routine physical examination was 42% (95%CI: 37% to 47%). One comprehensive survey of *H*. *pylori* infection from 2002 to 2004 in China reported that the average total infection rate of *H*. *pylori* was 56.22% [43]. Among the 22 studies we included, 21 were surveyed after 2007, indicating a decline in *H*. *pylori* infection rates in China. Another review also showed a decline in *H*. *pylori* infection rates in China up to 19 January 2015. [44] On the other hand, our results also suggests that *H*. *pylori* may be one of risk factors for obesity with pooled OR of 1.15 (95% CI: 1.09 to 1.22). Besides, the mean difference of *H*. *pylori*-positive BMI was higher than that of negative, and the *H*. *pylori* infection rate of obese people was higher than that of normal people, indicating that there was a positive correlation between *H*. *pylori* infection and obesity. Compared with *H*. *pylori*-negative group, the BMI, systolic blood pressure, diastolic blood pressure, triglycerides, total cholesterol and serum LDL cholesterol of the *H*. *pylori*-positive group were all enhanced, and the average HDL cholesterol decreased, which testify to a worse energy metabolism.

The precise mechanism underlying these findings is not well established. There is a number of potential factors which may be involved. (1) Ghrelin and leptin, gastrointestinal hormones, are both involved in metabolic control and energy balance. Ghrelin is produced in the stomach and can stimulate food intake. Leptin has an opposite effect. It was mainly synthesized in adipose tissue [45] and also produced by P cells of the gastric epithelium [46]. Studies reported lower serum leptin levels [47] and lower serum Ghrelin levels in *H*. *pylori*-positive patients [47, 48, 49]. Leptin can inhibit eating, and its reduction may be involved in excessive eating and obesity. While the decrease of plasma Ghrelin concentration represented a physiological adaptation to the positive energy balance associated with obesity [50]. (2) Insulin resistance is an important risk factor in lots of common metabolic disorders [51, 52]. *H*. *pylori* infection was found to have a potential role in promoting insulin resistance as observed in a Japanese study [53]. So, people with *H*. *pylori* infection may be more likely to get obesity. (3) Obesity may interact with *H*. *pylori* infection. Recently, growing evidence has implicated the intestinal immune system as an important contributor to metabolic disease including obesity [54]. It has been reported that the ability of monocytes to convert into macrophages was decreased in morbid obesity patients [55], which indicate that immune environment of obese people is more powerful for *H*. *pylori* survival. On the other hand, pre-adipocytes could develop phagocytic activity toward microorganisms as macrophage-like cells until they stop proliferating and differentiate into adipocytes [56], which suggests that *H*. *pylori* infection may stimulate the growth of adipose tissue to participate in the immune process.

Our result supports a positive relationship between *H*. *pylori* infection and obesity. But, studies included in this review are all the cross-sectional studies so this study cannot establish a causal relationship between them. In addition, this study has several limitations. First, studies included are unable to distinguish the *H*. *pylori* genotypes. *H*. *pylori* type I strains which expressing cagAand vacA are more virulent than type II strains and may bring more effect on metabolism. Studies have shown that the variation of the 3′ Region of the cagA Gene in *H*. *pylori* is closely related to the pathological changes and clinical outcomes caused by the infection of the strain [57]. But how these gene differences affect obesity remains unclear. Second, subjects in our study were people who took health examination from urban area, so we may underestimate the prevalence of *H*. *pylori* in China. More researches on *H*. *pylori* infection and obesity in rural areas are needed. Last, obesity is a chronic disease that is also affected by heredity and lifestyle. There are existing more than one major gene influencing BMI in Chinese sample [58]. In Chinese population, Genetic variation in the FTO gene is strongly associated with obesity and BMI, and its effect size on BMI is comparable with that in the European population [59]. NOC Gene, which is one of circadian clock genes, is also associated with obesity and BMI [60]. Besides, the AC3 genetic polymorphisms are associated with obesity in adults but not in children [61]. So, further studies are needed to control the confounders and to verify or strengthen the association. It would be important to note that the evaluation of the *H*. *pylori* infection rate in several subgroups and the calculation of pooled values were not determined by all studies but some subsets. Because only these subsets contains the data needed for the calculation. Notice that there was significant heterogeneity between studies when we compared the mean difference of biochemical characteristics, but hardly any heterogeneity when evaluated the risk of obesity for *H*. *pylori*-positive subjects, we thought this was due to the intrinsic properties of the object being analyzed. For the former’s heterogeneities, we considered difference in survey regions, *H*. *pylori* test methods, socioeconomic status of subjects, and other factors as sources of them.

*H*. *pylori*, a pathogenic bacteria of gastrointestinal diseases, has relationship with many extra-intestinal diseases. However, *H*. *pylori* is not on the adverse effects of all diseases. It presents a protective factor in the onset of certain diseases, which may inspire new diagnosis and therapeutic methods for obesity and other diseases.

## Supporting information

S1 PRISMA Checklist PRISMA 2009 Checklist. (DOC)
